# Supplementary material for: Carbapenemases on the move: it’s good to be on ICEs
Source: Mob DNA. 2018 Dec 19;9:37. doi: 10.1186/s13100-018-0141-4 (PMC6299553; doi:10.1186/s13100-018-0141-4)
Supplement: Supplementary file 1 — Table S1. General features of the hits. Hits associated with ICEs are highlighted in blue. Strains for which more than one CEG was identified are represented in red. (DOCX 52 kb) [file 13100_2018_141_MOESM1_ESM.docx]

**Table S1** – General features of the hits. Hits associated with ICEs are highlighted in blue. Strains for which more than one CEG was identified are represented in red.

| CEG | Accession number | Evalue | Bitscore | Organism | Strain | Sequence Length | Collection Date | Country | Isolation Source |
| --- | --- | --- | --- | --- | --- | --- | --- | --- | --- |
| DIM-1 | NZ_NXHR01000090.1 | 5.3e-147 | 513.5 | *Pseudomonas aeruginosa* | 97 | 3093 | 2015 | Ghana: Kumasi | Urine |
| DIM-1 | NZ_NXHP01000163.1 | 5.3e-147 | 513.5 | *Pseudomonas aeruginosa* | 130 | 3093 | 2015 | Ghana: Kumasi | Wound swab |
| DIM-1 | NZ_NXHO01000169.1 | 5.3e-147 | 513.5 | *Pseudomonas aeruginosa* | 140 | 3093 | 2015 | Ghana: Kumasi | Wound swab |
| DIM-1 | NZ_NXHN01000226.1 | 5.3e-147 | 513.5 | *Pseudomonas aeruginosa* | 142 | 3093 | 2015 | Ghana: Kumasi | Pleural fluid |
| DIM-1 | NZ_AP017302.1 | 2.6e-143 | 512.3 | *Pseudomonas aeruginosa* | IOMTU 133 | 6897018 | 2012 | Nepal: Kathmandu | Urinary catheter |
| FIM-1 | NZ_JTWC01000189.1 | 1.1e-154 | 538.5 | *Pseudomonas aeruginosa* | AZPAE14730 | 2154 | 2012 | Italy | Respiratory tract infection |
| FIM-1 | NZ_LYTT01000036.1 | 3.0e-154 | 538.5 | *Pseudomonas aeruginosa* | TRN6601 | 5964 | 2012 | USA | NA |
| GES-20 | NZ_JNHD01000009.1 | 7.8e-161 | 565.5 | *Pseudomonas aeruginosa* | PA_ST235 | 206205 | 2008 | Spain: Madrid | Blood |
| GES-20 | NZ_CP021774.1 | 2.7e-159 | 565.5 | *Pseudomonas aeruginosa* | Pa124 | 7008516 | 2006 | Mexico: Mexico city | Bronchial washing |
| GES-20 | NZ_CP022000.1 | 2.7e-159 | 565.5 | *Pseudomonas aeruginosa* | Pa127 | 7148302 | 2006 | Mexico: Mexico city | Bronchial washing |
| GES-5 | NZ_JTQT01000068.1 | 1.8e-161 | 567.0 | *Pseudomonas aeruginosa* | AZPAE14948 | 138245 | 2009 | Argentina: Victoria | Intra-abdominal tract infection |
| GES-5 | NZ_LFMR01000003.1 | 6.9e-161 | 567.0 | *Pseudomonas aeruginosa* | BTP034 | 527128 | 2014 | USA: Rochester, Minnesota | Associated Infection |
| GES-5 | NZ_PQGF01000119.1 | 2.1e-163 | 567.0 | *Pseudomonas aeruginosa* | Pa64 | 1603 | 2009 | Brazil | Soft tissue |
| GES-5 | NZ_LSZV01000002.1 | 2.9e-161 | 567.0 | *Pseudomonas aeruginosa* | RNS_PA1 | 223457 | 2006 | Australia: Sydney | Rectal swab |
| GES-5 | NZ_LVED01000149.1 | 6.1e-163 | 567.0 | *Pseudomonas aeruginosa* | RNS_PA69 | 4698 | 2007 | Australia: Sydney | Blood culture |
| GES-5 | NZ_LSZT01000004.1 | 9.1e-163 | 567.0 | *Pseudomonas aeruginosa* | RNS_PAE05 | 7001 | 2007 | Australia: Sydney | Hospital ward |
| GES-5 | NZ_LVEF01000109.1 | 6.1e-163 | 567.0 | *Pseudomonas aeruginosa* | RNS_PAE08 | 4698 | 2007 | Australia: Sydney | Hospital gel hand wash |
| GES-5 | NZ_NFGS01000071.1 | 6.4e-163 | 567.0 | *Pseudomonas aeruginosa* | S122_C02_RS | 4907 | 2013/2014 | Italy: Lecco | Respiratory Sample |
| GES-5 | NZ_NFGX01000057.1 | 6.4e-163 | 567.0 | *Pseudomonas aeruginosa* | S39_C01_BS | 4907 | 2013/2014 | Italy: Milano | Blood |
| GES-5 | NZ_NFGL01000183.1 | 5.8e-163 | 567.0 | *Pseudomonas aeruginosa* | S402_C09_RS | 4454 | 2013/2014 | Italy: Udine | Respiratory Sample |
| GES-5 | NZ_NFGW01000066.1 | 5.8e-163 | 567.0 | *Pseudomonas aeruginosa* | S49_C01_BS | 4454 | 2013/2014 | Italy: Milano | Blood |
| GES-5 | NZ_LYTY01000075.1 | 3.6e-163 | 567.0 | *Pseudomonas aeruginosa* | TRN6637 | 2779 | 2013 | Russia | NA |
| GES-6 | NZ_NBEZ01000053.1 | 4.4e-163 | 567.0 | *Pseudomonas aeruginosa* | FFUP_PS_690 | 3393 | 2015 | Portugal: Porto | Urine |
| IMP-1 | NZ_NXHR01000094.1 | 4.2e-145 | 506.9 | *Pseudomonas aeruginosa* | 97 | 2659 | 2015 | Ghana: Kumasi | Urine |
| IMP-1 | NZ_NXHP01000170.1 | 4.2e-145 | 506.9 | *Pseudomonas aeruginosa* | 130 | 2659 | 2015 | Ghana: Kumasi | Wound swab |
| IMP-1 | NZ_NXHO01000173.1 | 4.2e-145 | 506.9 | *Pseudomonas aeruginosa* | 140 | 2659 | 2015 | Ghana: Kumasi | Wound swab |
| IMP-1 | NZ_NXHN01000235.1 | 4.2e-145 | 506.9 | *Pseudomonas aeruginosa* | 142 | 2659 | 2015 | Ghana: Kumasi | Pleural fluid |
| IMP-1 | NZ_CP024477.1 | 1.1e-141 | 506.9 | *Pseudomonas aeruginosa* | 12939 | 6621378 | 2013 | China | Medium |
| IMP-1 | NZ_MPBP01000001.1 | 1.6e-141 | 506.1 | *Pseudomonas aeruginosa* | AR_0103 | 5781139 | NA | NA | NA |
| IMP-1 | NZ_MPBP01000001.1 | 1.6e-141 | 506.1 | *Pseudomonas aeruginosa* | AR_0103 | 5781139 | NA | NA | NA |
| IMP-1 | NZ_BCAQ01000182.1 | 5.2e-145 | 506.5 | *Pseudomonas sp.* | GTC 16473 | 2488 | NA | NA | NA |
| IMP-1 | NZ_BCAS01000276.1 | 3.1e-145 | 505.8 | *Pseudomonas sp.* | GTC 16482 | 875 | NA | NA | NA |
| IMP-1 | NZ_BCAV01000087.1 | 1.5e-144 | 506.1 | *Pseudomonas sp.* | NBRC 111119 | 5517 | NA | NA | NA |
| IMP-1 | NZ_BCAX01000074.1 | 4.7e-144 | 506.1 | *Pseudomonas sp.* | NBRC 111121 | 17324 | NA | NA | NA |
| IMP-1 | NZ_BCBC01000204.1 | 3.1e-145 | 505.8 | *Pseudomonas sp.* | NBRC 111126 | 874 | NA | NA | NA |
| IMP-1 | NZ_BCBF01000218.1 | 1.9e-145 | 506.5 | *Pseudomonas sp.* | NBRC 111129 | 888 | NA | NA | NA |
| IMP-1 | NZ_BCBU01000102.1 | 5.0e-144 | 506.1 | *Pseudomonas sp.* | NBRC 111144 | 18180 | NA | NA | NA |
| IMP-1 | NZ_DF126593.1 | 1.9e-141 | 506.1 | *Pseudomonas aeruginosa* | NCGM1179 | 6910294 | NA | NA | NA |
| IMP-1 | NC_017549.1 | 1.8e-141 | 506.1 | *Pseudomonas aeruginosa* | NCGM2.S1 | 6764661 | NA | NA | NA |
| IMP-1 | NZ_AP014651.1 | 1.9e-141 | 506.1 | *Pseudomonas aeruginosa* | NCGM257 | 7090694 | 2014 | NA | Midstream urine |
| IMP-1 | NZ_CVUY01000246.1 | 3.3e-143 | 506.5 | *Pseudomonas aeruginosa* | P1_London_28_IMP_1_04_05 | 158152 | NA | NA | NA |
| IMP-1 | NZ_CVWC01000363.1 | 7.9e-144 | 506.5 | *Pseudomonas aeruginosa* | P2_London_28_IMP_1_06_05 | 37866 | NA | NA | NA |
| IMP-1 | NZ_CWFP01000391.1 | 5.3e-144 | 506.5 | *Pseudomonas aeruginosa* | P6_East_of_England_6_IMP_1_03_09 | 25204 | NA | NA | NA |
| IMP-1 | NZ_LYTV01000132.1 | 8.0e-145 | 506.1 | *Pseudomonas aeruginosa* | TRN6622 | 2924 | 2012 | Thailand | NA |
| IMP-10 | NZ_BCBB01000121.1 | 3.2e-144 | 506.9 | *Pseudomonas sp.* | NBRC 111125 | 20110 | NA | NA | NA |
| IMP-10 | NZ_BCBS01000290.1 | 2.5e-145 | 507.3 | *Pseudomonas sp.* | NBRC 111142 | 2076 | NA | NA | NA |
| IMP-10 | NZ_BCBT01000045.1 | 6.9e-144 | 507.3 | *Pseudomonas sp.* | NBRC 111143 | 56542 | NA | NA | NA |
| IMP-13 | NZ_JTUA01000030.1 | 6.2e-143 | 505.4 | *Pseudomonas aeruginosa* | AZPAE14862 | 133844 | 2007 | India: Chennai | Urinary tract infection |
| IMP-13 | NZ_CVUU01000081.1 | 2.7e-145 | 506.1 | *Pseudomonas aeruginosa* | P23_East_of_England_6_IMP_13_07_10 | 987 | NA | NA | NA |
| IMP-13 | NZ_CWEU01000115.1 | 2.7e-145 | 506.1 | *Pseudomonas aeruginosa* | P9_East_of_England_6_IMP_13_08_09 | 987 | NA | NA | NA |
| IMP-13 | NZ_NFGR01000020.1 | 6.2e-143 | 505.4 | *Pseudomonas aeruginosa* | S137_C02_RS | 134100 | 2013/2014 | Italy: Lecco | Respiratory Sample |
| IMP-13 | NZ_NFGQ01000074.1 | 2.4e-144 | 505.4 | *Pseudomonas aeruginosa* | S143_C02_RS | 5135 | 2013/2014 | Italy: Lecco | Respiratory Sample |
| IMP-13 | NZ_NFGP01000019.1 | 6.2e-143 | 505.4 | *Pseudomonas aeruginosa* | S220_C06_RS | 134100 | 2013/2014 | Italy: Modena | Respiratory Sample |
| IMP-13 | NZ_NFGO01000041.1 | 1.8e-143 | 505.4 | *Pseudomonas aeruginosa* | S247_C06_RS | 38967 | 2013/2014 | Italy: Modena | Respiratory Sample |
| IMP-13 | NZ_NFFS01000020.1 | 6.2e-143 | 505.4 | *Pseudomonas aeruginosa* | S668_C14_BS | 134100 | 2013/2014 | Italy: Roma | Blood |
| IMP-13 | NZ_NFFQ01000021.1 | 6.2e-143 | 505.4 | *Pseudomonas aeruginosa* | S700_C14_RS | 134100 | 2013/2014 | Italy: Roma | Respiratory Sample |
| IMP-13 | NZ_NFFM01000067.1 | 1.8e-144 | 505.4 | *Pseudomonas aeruginosa* | S769_C16_RS | 3873 | 2013/2014 | Italy: Casarano | Respiratory Sample |
| IMP-13 | NZ_NFFL01000020.1 | 6.2e-143 | 505.4 | *Pseudomonas aeruginosa* | S782_C16_RS | 134000 | 2013/2014 | Italy: Casarano | Respiratory Sample |
| IMP-13 | NZ_NFFK01000022.1 | 6.2e-143 | 505.4 | *Pseudomonas aeruginosa* | S787_C16_RS | 134000 | 2013/2014 | Italy: Casarano | Respiratory Sample |
| IMP-13 | NZ_NFFI01000020.1 | 6.2e-143 | 505.4 | *Pseudomonas aeruginosa* | S811_C17_BS | 134000 | 2013/2014 | Italy: Napoli | Blood |
| IMP-13 | NZ_NFFE01000043.1 | 1.8e-143 | 505.4 | *Pseudomonas aeruginosa* | S829_C17_RS | 38725 | 2013/2014 | Italy: Napoli | Respiratory Sample |
| IMP-14 | NZ_MPBS01000001.1 | 7.2e-141 | 504.2 | *Pseudomonas aeruginosa* | AR_0092 | 6963676 | NA | NA | NA |
| IMP-14 | NZ_JXBE01000114.1 | 1.6e-144 | 504.2 | *Pseudomonas aeruginosa* | ST260 | 1535 | 2012 | Australia: Victoria | Blood culture |
| IMP-15 | NZ_JTZG01000017.1 | 1.9e-145 | 511.9 | *Pseudomonas aeruginosa* | AZPAE13872 | 38616 | 2010 | Mexico | NA |
| IMP-18 | NZ_JTZQ01000083.1 | 8.0e-144 | 503.1 | *Pseudomonas aeruginosa* | AZPAE13756 | 3455 | 2009 | Canada | Respiratory tract infection |
| IMP-18 | NZ_CVVU01000084.1 | 6.5e-144 | 503.1 | *Pseudomonas aeruginosa* | P19_London_7_VIM_2_05_10 | 2825 | NA | NA | NA |
| IMP-19 | NZ_NFFT01000053.1 | 5.5e-145 | 507.3 | *Pseudomonas aeruginosa* | S658_C13_RS | 4525 | 2013/2014 | Italy: Ancona | Respiratory Sample |
| IMP-26 | NZ_JTXD01000152.1 | 1.5e-145 | 508.4 | *Pseudomonas aeruginosa* | AZPAE14702 | 2812 | 2012 | Philippines | Respiratory tract infection |
| IMP-26 | NZ_NIJE01000027.1 | 4.6e-144 | 508.4 | *Pseudomonas aeruginosa* | PAS1 | 84097 | 2009 | Malaysia: Kuala Lumpur | Wound |
| IMP-26 | NZ_NIJB01000026.1 | 6.3e-144 | 508.1 | *Pseudomonas aeruginosa* | PAS4 | 87244 | 2009 | Malaysia: Kuala Lumpur | Urine |
| IMP-26 | NZ_NIIY01000031.1 | 4.6e-144 | 508.4 | *Pseudomonas aeruginosa* | PAS7 | 83381 | 2010 | Malaysia: Kuala Lumpur | Urine |
| IMP-34 | NZ_AP014622.1 | 8.3e-142 | 507.3 | *Pseudomonas aeruginosa* | NCGM 1900 | 6814936 | NA | NA | NA |
| IMP-34 | NZ_AP014622.1 | 8.3e-142 | 507.3 | *Pseudomonas aeruginosa* | NCGM 1900 | 6814936 | NA | NA | NA |
| IMP-34 | NZ_AP014646.1 | 8.4e-142 | 507.3 | *Pseudomonas aeruginosa* | NCGM 1984 | 6850954 | NA | NA | NA |
| IMP-34 | NZ_AP014646.1 | 8.4e-142 | 507.3 | *Pseudomonas aeruginosa* | NCGM 1984 | 6850954 | NA | NA | NA |
| IMP-45 | NZ_MKEO01001262.1 | 8.3e-147 | 512.3 | *Pseudomonas aeruginosa* | M140A | 2177 | 2012 | China: Guangzhou, Guangdong | Urine |
| IMP-45 | NZ_CP016215.1 | 1.6e-144 | 512.3 | *Pseudomonas aeruginosa* | PA121617 | 423017 | 2012 | China: Guangzhou | Sputum |
| IMP-45 | NZ_MKEM01000369.1 | 1.5e-146 | 512.3 | *Pseudomonas aeruginosa* | PA13SY16 | 3876 | 2013 | China: Guangzhou, Guangdong | Urine |
| IMP-45 | NZ_MBPN01000205.1 | 1.5e-146 | 512.3 | *Pseudomonas sp.* | WCHP16 | 3842 | 2015 | China: Sichuan | Sewage |
| IMP-56 | NZ_JTXQ01000099.1 | 1.2e-144 | 504.6 | *Pseudomonas aeruginosa* | AZPAE14688 | 1534 | 2012 | Mexico | NA |
| IMP-62 | NZ_JTXR01000035.1 | 7.3e-147 | 513.1 | *Pseudomonas aeruginosa* | AZPAE14687 | 3272 | 2012 | Mexico | Respiratory tract infection |
| IMP-7 | NZ_MPCR01000099.1 | 3.8e-145 | 506.1 | *Pseudomonas aeruginosa* | PAC17 | 1380 | 2014 | Malaysia | Blood |
| IMP-7 | NZ_MWWM01000109.1 | 4.2e-145 | 506.9 | *Pseudomonas aeruginosa* | UQCCR 393788042 K AB94 | 2644 | 2009 | Australia: Brisbane | Blood |
| IMP-8 | NZ_MJMC01000004.1 | 1.3e-144 | 509.2 | *Pseudomonas aeruginosa* | PA77 | 39882 | 2010 | Germany | NA |
| IMP-9 | NC_022344.1 | 5.5e-144 | 510.8 | *Pseudomonas aeruginosa* | PA96 | 500839 | NA | NA | NA |
| KPC-2 | NZ_LODN01000080.1 | 1.0e-166 | 582.4 | *Pseudomonas aeruginosa* | AATYA | 33551 | 2014 | USA | NA |
| KPC-2 | NZ_LQXW01000026.1 | 1.8e-166 | 582.4 | *Pseudomonas sp.* | ABFPK | 59517 | 2014 | USA | NA |
| KPC-2 | NZ_CP027168.1 | 1.7e-166 | 582.4 | *Pseudomonas aeruginosa* | AR_0356 | 57053 | NA | NA | NA |
| KPC-2 | NZ_JTWN01000034.1 | 3.5e-167 | 582.4 | *Pseudomonas aeruginosa* | AZPAE14719 | 11794 | 2012 | Colombia | Respiratory tract infection |
| KPC-2 | NZ_JTWM01000123.1 | 3.5e-167 | 582.4 | *Pseudomonas aeruginosa* | AZPAE14720 | 11794 | 2012 | Colombia | Urinary tract infection |
| KPC-2 | NZ_JTWL01000087.1 | 5.1e-167 | 582.4 | *Pseudomonas aeruginosa* | AZPAE14721 | 17094 | 2012 | Colombia | Intra-abdominal tract infection |
| KPC-2 | NZ_NOKO01000029.1 | 3.4e-167 | 582.4 | *Pseudomonas aeruginosa* | CCBH17348 | 11227 | 2014 | Brazil | Blood |
| KPC-2 | NZ_CP026386.1 | 1.9e-164 | 582.4 | *Pseudomonas sp.* | PONIH3 | 6313552 | 2014 | USA | NA |
| KPC-2 | NZ_CP026386.1 | 1.9e-164 | 582.4 | *Pseudomonas sp.* | PONIH3 | 6313552 | 2014 | USA | NA |
| NDM-1 | NZ_PESJ01000063.1 | 3.1e-156 | 544.7 | *Pseudomonas sp.* | MR 02 | 4495 | 2016 | India | River |
| NDM-1 | NZ_CP020703.1 | 4.8e-153 | 544.7 | *Pseudomonas aeruginosa* | PASGNDM345 | 6893164 | 2015 | Singapore | Sputum |
| NDM-1 | NZ_NDFQ01000052.1 | 1.6e-156 | 544.7 | *Pseudomonas aeruginosa* | PASGNDM544 | 2365 | 2015 | Singapore | Endotracheal tube (ETT) aspirate |
| NDM-1 | NZ_NDFR01000042.1 | 1.6e-156 | 544.7 | *Pseudomonas aeruginosa* | PASGNDM571 | 2365 | 2015 | Singapore | Urine |
| NDM-1 | NZ_NDFS01000048.1 | 1.8e-156 | 544.7 | *Pseudomonas aeruginosa* | PASGNDM583 | 2597 | 2015 | Singapore | Urine |
| NDM-1 | NZ_NDFT01000055.1 | 1.8e-156 | 544.7 | *Pseudomonas aeruginosa* | PASGNDM586 | 2597 | 2015 | Singapore | Urine |
| NDM-1 | NZ_NDFV01000059.1 | 1.6e-156 | 544.7 | *Pseudomonas aeruginosa* | PASGNDM587 | 2365 | 2015 | Singapore | Foot wound swab |
| NDM-1 | NZ_NDFU01000049.1 | 1.6e-156 | 544.7 | *Pseudomonas aeruginosa* | PASGNDM590 | 2310 | 2015 | Singapore | Urine |
| NDM-1 | NZ_NDFW01000065.1 | 1.6e-156 | 544.7 | *Pseudomonas aeruginosa* | PASGNDM591 | 2365 | 2015 | Singapore | Urine |
| NDM-1 | NZ_NDFX01000043.1 | 1.6e-156 | 544.7 | *Pseudomonas aeruginosa* | PASGNDM592 | 2365 | 2015 | Singapore | Urine |
| NDM-1 | NZ_NDFY01000046.1 | 1.6e-156 | 544.7 | *Pseudomonas aeruginosa* | PASGNDM593 | 2365 | 2015 | Singapore | Urine |
| NDM-1 | NZ_CP020704.1 | 4.8e-153 | 544.7 | *Pseudomonas aeruginosa* | PASGNDM699 | 6985102 | 2015 | Singapore | Sputum |
| SPM-1 | NZ_AFXJ01000001.1 | 2.2e-158 | 562.4 | *Pseudomonas aeruginosa* | 19BR | 6742964 | NA | NA | NA |
| SPM-1 | NZ_AFXK01000001.1 | 2.2e-158 | 562.4 | *Pseudomonas aeruginosa* | 213BR | 6719211 | NA | NA | NA |
| SPM-1 | NZ_AFXI01000001.1 | 2.2e-158 | 562.4 | *Pseudomonas aeruginosa* | 9BR | 6801503 | NA | NA | NA |
| SPM-1 | NZ_MPBU01000007.1 | 7.0e-159 | 562.4 | *Pseudomonas aeruginosa* | AR_0064 | 2188636 | NA | NA | NA |
| SPM-1 | NZ_JTVP01000053.1 | 4.5e-162 | 562.4 | *Pseudomonas aeruginosa* | AZPAE14819 | 1419 | 2004 | Brazil: Sao Paulo | Urinary tract infection |
| SPM-1 | NZ_JTVN01000070.1 | 4.5e-162 | 562.4 | *Pseudomonas aeruginosa* | AZPAE14821 | 1419 | 2004 | Brazil: Sao Paulo | Urinary tract infection |
| SPM-1 | NZ_JTUI01000094.1 | 4.5e-162 | 562.4 | *Pseudomonas aeruginosa* | AZPAE14853 | 1417 | 2007 | Brazil: Curitiba | Respiratory tract infection |
| SPM-1 | NZ_JTRS01000063.1 | 4.5e-162 | 562.4 | *Pseudomonas aeruginosa* | AZPAE14923 | 1417 | 2008 | Brazil: Sao Paulo | Respiratory tract infection |
| SPM-1 | NZ_CP021380.1 | 2.3e-158 | 562.4 | *Pseudomonas aeruginosa* | CCBH4851 | 7060875 | 2008 | Brazil | Catheter tip |
| SPM-1 | NZ_CP021380.1 | 2.3e-158 | 562.4 | *Pseudomonas aeruginosa* | CCBH4851 | 7060875 | 2008 | Brazil | Catheter tip |
| SPM-1 | NZ_CP015001.1 | 2.2e-158 | 562.4 | *Pseudomonas aeruginosa* | PA1088 | 6721480 | 1997 | Brazil: Sao Paulo, SP | Urine |
| SPM-1 | NZ_CP015003.1 | 2.2e-158 | 562.4 | *Pseudomonas aeruginosa* | PA11803 | 7006578 | 2011 | Brazil: Sao Paulo, SP | Bloodstream |
| SPM-1 | NZ_LVXB01000001.1 | 1.1e-158 | 562.4 | *Pseudomonas aeruginosa* | PA12117 | 3316996 | 2012 | Brazil: Sao Paulo, SP | Bloodstream |
| SPM-1 | NZ_PHSS01000091.1 | 4.2e-162 | 562.4 | *Pseudomonas aeruginosa* | PA151 | 1306 | 2011 | Brazil: Sao Paulo | River |
| SPM-1 | NZ_PHST01000144.1 | 1.1e-161 | 562.4 | *Pseudomonas aeruginosa* | PA19 | 3393 | 2010 | Brazil: Sao Paulo | River |
| SPM-1 | NZ_LVWC01000001.1 | 2.2e-158 | 562.4 | *Pseudomonas aeruginosa* | PA3448 | 6788539 | 2003 | Brazil: Sao Paulo -SP | bloodstream |
| SPM-1 | NZ_LVWC01000001.1 | 2.2e-158 | 562.4 | *Pseudomonas aeruginosa* | PA3448 | 6788539 | 2003 | Brazil: Sao Paulo -SP | Bloodstream |
| SPM-1 | NZ_CP014999.1 | 2.2e-158 | 562.4 | *Pseudomonas aeruginosa* | PA7790 | 7018690 | 2006 | Brazil: Sao Paulo, SP | Tracheal aspirate |
| SPM-1 | NZ_CP015002.1 | 2.2e-158 | 562.4 | *Pseudomonas aeruginosa* | PA8281 | 6928736 | 2007 | Brazil: Sao Paulo, SP | Tracheal aspirate |
| SPM-1 | NZ_CP015002.1 | 2.2e-158 | 562.4 | *Pseudomonas aeruginosa* | PA8281 | 6928736 | 2007 | Brazil: Sao Paulo, SP | Tracheal aspirate |
| VIM-1 | NC_019906.1 | 1.1e-150 | 530.4 | *Pseudomonas putida* | HB3267 | 80360 | NA | NA | NA |
| VIM-1 | NZ_NFGN01000084.1 | 3.6e-152 | 530.4 | *Pseudomonas aeruginosa* | S252_C06_RS | 2641 | 2013/2014 | Italy: Modena | Respiratory Sample |
| VIM-1 | NZ_NFGM01000055.1 | 3.4e-152 | 530.4 | *Pseudomonas aeruginosa* | S292_C06_RS | 2541 | 2013/2014 | Italy: Modena | Respiratory Sample |
| VIM-1 | NZ_NFGC01000059.1 | 1.3e-151 | 530.4 | *Pseudomonas aeruginosa* | S461_C10_RS | 9365 | 2013/2014 | Italy: Firenze | Respiratory Sample |
| VIM-1 | NZ_NFGB01000058.1 | 4.8e-152 | 530.4 | *Pseudomonas aeruginosa* | S518_C10_BS | 3551 | 2013/2014 | Italy: Firenze | Blood |
| VIM-1 | NZ_NFGV01000057.1 | 2.2e-151 | 530.4 | *Pseudomonas aeruginosa* | S53_C01_BS | 16165 | 2013/2014 | Italy: Milano | Blood |
| VIM-1 | NZ_NFFZ01000073.1 | 4.4e-152 | 530.4 | *Pseudomonas aeruginosa* | S567_C10_BS | 3236 | 2013/2014 | Italy: Firenze | Blood |
| VIM-1 | NZ_NFFZ01000073.1 | 4.4e-152 | 530.4 | *Pseudomonas aeruginosa* | S567_C10_BS | 3236 | 2013/2014 | Italy: Firenze | Blood |
| VIM-1 | NZ_NFGU01000072.1 | 2.2e-151 | 530.4 | *Pseudomonas aeruginosa* | S57_C01_BS | 16191 | 2013/2014 | Italy: Milano | Blood |
| VIM-1 | NZ_NFFY01000029.1 | 1.1e-150 | 530.4 | *Pseudomonas aeruginosa* | S611_C13_RS | 78563 | 2013/2014 | Italy: Ancona | Respiratory Sample |
| VIM-1 | NZ_NFFX01000040.1 | 4.7e-151 | 530.4 | *Pseudomonas aeruginosa* | S619_C13_RS | 35200 | 2013/2014 | Italy: Ancona | Respiratory Sample |
| VIM-1 | NZ_NFFV01000038.1 | 6.5e-151 | 530.4 | *Pseudomonas aeruginosa* | S626_C13_RS | 48362 | 2013/2014 | Italy: Ancona | Respiratory Sample |
| VIM-1 | NZ_NFFU01000013.1 | 2.8e-150 | 530.4 | *Pseudomonas aeruginosa* | S650_C13_BS | 207944 | 2013/2014 | Italy: Ancona | Blood |
| VIM-1 | NZ_NFFO01000062.1 | 4.0e-151 | 530.4 | *Pseudomonas aeruginosa* | S742_C15_BS | 29466 | 2013/2014 | Italy: San Giovanni Rotondo | Blood |
| VIM-1 | NZ_NFFG01000090.1 | 6.5e-152 | 530.4 | *Pseudomonas aeruginosa* | S823_C17_RS | 4788 | 2013/2014 | Italy: Napoli | Respiratory Sample |
| VIM-1 | NZ_NFFC01000079.1 | 5.1e-152 | 530.4 | *Pseudomonas aeruginosa* | S854_C18_BS | 3763 | 2013/2014 | Italy: Cosenza | Blood |
| VIM-11 | NZ_JTZD01000113.1 | 8.4e-152 | 528.5 | *Pseudomonas aeruginosa* | AZPAE13879 | 1646 | 2010 | Argentina | NA |
| VIM-11 | NZ_JYGC02000004.1 | 2.6e-149 | 528.9 | *Pseudomonas aeruginosa* | MRSN 20176 | 657351 | 2013 | Afghanistan | Surveillance swab |
| VIM-2 | NZ_MWUI01000032.1 | 8.9e-151 | 530.0 | *Pseudomonas stutzeri* | 40D2 | 50445 | 2012 | Bangladesh: Dhaka | Inflamed tissue lesion of infected patient during surgery |
| VIM-2 | NZ_MPBQ01000001.1 | 7.7e-149 | 530.0 | *Pseudomonas aeruginosa* | AR_0100 | 4354749 | NA | NA | NA |
| VIM-2 | NZ_MPBN01000001.1 | 1.2e-148 | 530.0 | *Pseudomonas aeruginosa* | AR_0108 | 6879367 | NA | NA | NA |
| VIM-2 | NZ_CP027174.1 | 1.2e-148 | 530.0 | *Pseudomonas aeruginosa* | AR_0230 | 7012922 | NA | NA | NA |
| VIM-2 | NZ_JTZQ01000101.1 | 1.7e-152 | 530.0 | *Pseudomonas aeruginosa* | AZPAE13756 | 973 | 2009 | Canada | Respiratory tract infection |
| VIM-2 | NZ_JTZM01000008.1 | 9.4e-150 | 530.0 | *Pseudomonas aeruginosa* | AZPAE13853 | 531755 | 2010 | India | NA |
| VIM-2 | NZ_JTZK01000020.1 | 3.3e-150 | 530.0 | *Pseudomonas aeruginosa* | AZPAE13858 | 185470 | 2010 | India | NA |
| VIM-2 | NZ_JTZE01000060.1 | 3.4e-152 | 530.0 | *Pseudomonas aeruginosa* | AZPAE13877 | 1932 | 2010 | Romania | NA |
| VIM-2 | NZ_JTYE01000112.1 | 1.7e-152 | 530.0 | *Pseudomonas aeruginosa* | AZPAE14463 | 974 | 2011 | Colombia: Bogota | Urinary tract infection |
| VIM-2 | NZ_JTXF01000066.1 | 1.4e-150 | 530.0 | *Pseudomonas aeruginosa* | AZPAE14700 | 81597 | 2012 | Philippines | Respiratory tract infection |
| VIM-2 | NZ_JTXC01000105.1 | 4.0e-152 | 530.0 | *Pseudomonas aeruginosa* | AZPAE14703 | 2264 | 2012 | Philippines | Intra-abdominal tract infection |
| VIM-2 | NZ_JTXA01000187.1 | 4.0e-152 | 530.0 | *Pseudomonas aeruginosa* | AZPAE14705 | 2264 | 2012 | Greece | Urinary tract infection |
| VIM-2 | NZ_JTWY01000131.1 | 1.7e-152 | 530.0 | *Pseudomonas aeruginosa* | AZPAE14707 | 971 | 2012 | Greece | Respiratory tract infection |
| VIM-2 | NZ_JTWU01000220.1 | 1.7e-152 | 530.0 | *Pseudomonas aeruginosa* | AZPAE14712 | 943 | 2012 | Venezuela | Intra-abdominal tract infection |
| VIM-2 | NZ_JTWT01000168.1 | 1.7e-152 | 530.0 | *Pseudomonas aeruginosa* | AZPAE14713 | 972 | 2012 | Venezuela | Intra-abdominal tract infection |
| VIM-2 | NZ_JTWS01000232.1 | 1.7e-152 | 530.0 | *Pseudomonas aeruginosa* | AZPAE14714 | 972 | 2012 | Venezuela | Intra-abdominal tract infection |
| VIM-2 | NZ_JTWQ01000139.1 | 3.2e-152 | 530.0 | *Pseudomonas aeruginosa* | AZPAE14716 | 1814 | 2012 | Venezuela | Intra-abdominal tract infection |
| VIM-2 | NZ_JTWO01000202.1 | 4.4e-152 | 530.0 | *Pseudomonas aeruginosa* | AZPAE14718 | 2520 | 2012 | USA | Respiratory tract infection |
| VIM-2 | NZ_JTWI01000104.1 | 1.7e-152 | 530.0 | *Pseudomonas aeruginosa* | AZPAE14724 | 972 | 2012 | Italy | Intra-abdominal tract infection |
| VIM-2 | NZ_JTWD01000173.1 | 1.7e-152 | 530.0 | *Pseudomonas aeruginosa* | AZPAE14729 | 972 | 2012 | Italy | Urinary tract infection |
| VIM-2 | NZ_JTVX01000068.1 | 1.1e-151 | 530.0 | *Pseudomonas aeruginosa* | AZPAE14811 | 6396 | 2004 | India: Mumbai | Respiratory tract infection |
| VIM-2 | NZ_JTRT01000042.1 | 7.2e-152 | 530.0 | *Pseudomonas aeruginosa* | AZPAE14922 | 4067 | 2009 | France: Paris | Respiratory tract infection |
| VIM-2 | NZ_JTRM01000079.1 | 3.4e-152 | 530.0 | *Pseudomonas aeruginosa* | AZPAE14929 | 1932 | 2009 | Germany: Aachen | Urinary tract infection |
| VIM-2 | NZ_JTQI01000054.1 | 3.5e-152 | 530.4 | *Pseudomonas aeruginosa* | AZPAE14959 | 2614 | 2009 | India: Mumbai | Intra-abdominal tract infection |
| VIM-2 | NZ_JTPK01000055.1 | 7.2e-152 | 530.0 | *Pseudomonas aeruginosa* | AZPAE14984 | 4068 | 2010 | France: Paris | Urinary tract infection |
| VIM-2 | NZ_JTNR01000060.1 | 2.3e-151 | 530.0 | *Pseudomonas aeruginosa* | AZPAE15029 | 12782 | 2011 | France: Paris | Respiratory tract infection |
| VIM-2 | NZ_LFDH01000007.1 | 4.0e-150 | 530.0 | *Pseudomonas aeruginosa* | BK6 | 227447 | 2013 | India: Madurai | Cornea from keratitis patient |
| VIM-2 | NZ_LFMP01000001.1 | 4.9e-149 | 530.0 | *Pseudomonas aeruginosa* | BTP032 | 2771368 | 2014 | USA: Rochester, Minnesota | Associated Infection |
| VIM-2 | NZ_LFMV01000038.1 | 1.5e-149 | 530.0 | *Pseudomonas aeruginosa* | BTP038 | 838992 | 2014 | USA: Rochester, Minnesota | Associated Infection |
| VIM-2 | NZ_CP011317.1 | 1.3e-148 | 530.0 | *Pseudomonas aeruginosa* | Carb01 63 | 7497593 | 2012 | Netherlands:Rotterdam | Microbial feature |
| VIM-2 | NZ_NBVZ01000065.1 | 1.7e-151 | 530.4 | *Pseudomonas aeruginosa* | DZ-B1 | 12785 | 2014 | China: Shandong | Feces swab |
| VIM-2 | NZ_CWGH01000042.1 | 1.6e-151 | 530.0 | *Pseudomonas aeruginosa* | E1_London_17_VIM_2_02_09 | 9036 | NA | NA | NA |
| VIM-2 | NZ_CVVZ01000076.1 | 1.7e-151 | 530.0 | *Pseudomonas aeruginosa* | E10_London_26_VIM_2_06_13 | 9854 | NA | NA | NA |
| VIM-2 | NZ_CVVM01000189.1 | 2.6e-151 | 530.0 | *Pseudomonas aeruginosa* | E11_London_26_VIM_2_06_13 | 15035 | NA | NA | NA |
| VIM-2 | NZ_CVUX01000007.1 | 2.2e-151 | 530.0 | *Pseudomonas aeruginosa* | E12_London_26_VIM_2_06_13 | 12608 | NA | NA | NA |
| VIM-2 | NZ_CVVQ01000050.1 | 2.8e-152 | 530.0 | *Pseudomonas aeruginosa* | E13_London_26_VIM_2_06_13 | 1571 | NA | NA | NA |
| VIM-2 | NZ_CWGG01000041.1 | 1.8e-152 | 530.0 | *Pseudomonas aeruginosa* | E14_London_26_VIM_2_06_13 | 1017 | NA | NA | NA |
| VIM-2 | NZ_CVWG01001514.1 | 6.0e-151 | 530.0 | *Pseudomonas aeruginosa* | E16_London_17_VIM_2_04_14 | 33943 | NA | NA | NA |
| VIM-2 | NZ_CVUZ01000153.1 | 2.8e-152 | 530.0 | *Pseudomonas aeruginosa* | E2_London_17_VIM_2_02_09 | 1571 | NA | NA | NA |
| VIM-2 | NZ_CVVB01000030.1 | 1.6e-151 | 530.0 | *Pseudomonas aeruginosa* | E3_London_17_VIM_2_03_09 | 9035 | NA | NA | NA |
| VIM-2 | NZ_CVUK01000042.1 | 1.8e-152 | 530.0 | *Pseudomonas aeruginosa* | E4_London_17_VIM_2_03_09 | 1020 | NA | NA | NA |
| VIM-2 | NZ_CVWE01000645.1 | 1.8e-152 | 530.0 | *Pseudomonas aeruginosa* | E5_London_17_VIM_2_12_12 | 1012 | NA | NA | NA |
| VIM-2 | NZ_CVWD01000239.1 | 1.8e-152 | 530.0 | *Pseudomonas aeruginosa* | E6_London_17_VIM_2_12_12 | 1002 | NA | NA | NA |
| VIM-2 | NZ_CVWB01000082.1 | 1.8e-151 | 530.0 | *Pseudomonas aeruginosa* | E7_London_9_VIM_2_02_13 | 10104 | NA | NA | NA |
| VIM-2 | NZ_CVUW01000087.1 | 1.8e-152 | 530.0 | *Pseudomonas aeruginosa* | E8_London_17_VIM_2_04_13 | 1009 | NA | NA | NA |
| VIM-2 | NZ_CVUN01000192.1 | 2.6e-151 | 530.0 | *Pseudomonas aeruginosa* | E9_London_17_VIM_2_04_13 | 15035 | NA | NA | NA |
| VIM-2 | NZ_NINS01000056.1 | 6.0e-151 | 530.0 | *Pseudomonas aeruginosa* | FFUP_PS_105 | 33810 | 2012 | Portugal | Urine |
| VIM-2 | NZ_NINQ01000001.1 | 5.4e-151 | 530.0 | *Pseudomonas aeruginosa* | FFUP_PS_12 | 30772 | 2002 | Portugal | NA |
| VIM-2 | NZ_NINR01000084.1 | 4.8e-151 | 530.0 | *Pseudomonas aeruginosa* | FFUP_PS_144 | 27056 | 2013 | Portugal | Urine |
| VIM-2 | NZ_NINU01000134.1 | 1.2e-151 | 529.3 | *Pseudomonas aeruginosa* | FFUP_PS_35 | 4035 | 2002 | Portugal | Urine |
| VIM-2 | NZ_NINT01000013.1 | 1.5e-150 | 530.0 | *Pseudomonas aeruginosa* | FFUP_PS_37 | 87430 | 2008 | Portugal | Bronchial secretions |
| VIM-2 | NZ_PJQP01000061.1 | 4.9e-152 | 530.0 | *Pseudomonas sp.* | FFUP_PS_41 | 2763 | 2008 | Portugal: Porto | Endotracheal tube secretions |
| VIM-2 | NZ_NINN01000021.1 | 1.7e-150 | 530.0 | *Pseudomonas aeruginosa* | FFUP_PS_65 | 99153 | 2010 | Portugal | Urine |
| VIM-2 | NZ_NINP01000047.1 | 4.7e-151 | 530.0 | *Pseudomonas aeruginosa* | FFUP_PS_CB5 | 26506 | 2002 | Portugal | Bronchial aspirate |
| VIM-2 | NZ_NINO01000017.1 | 5.7e-151 | 530.0 | *Pseudomonas aeruginosa* | FFUP_PS_CB58 | 32345 | 2004 | Portugal | Urine |
| VIM-2 | NZ_AEVV03000013.1 | 5.4e-151 | 530.0 | *Pseudomonas aeruginosa* | HB13 | 30558 | NA | NA | NA |
| VIM-2 | NZ_NINV01000065.1 | 2.7e-151 | 530.0 | *Pseudomonas aeruginosa* | HSV3483 | 15428 | 1995 | Portugal | Urine |
| VIM-2 | NZ_NWBV01000195.1 | 3.5e-152 | 530.0 | *Pseudomonas aeruginosa* | ICBBVIM-2 | 1962 | 2017 | Brazil: Sao Paulo | Oral swab |
| VIM-2 | NZ_MZND01000183.1 | 1.7e-152 | 530.0 | *Pseudomonas aeruginosa* | ICBDVIM-2 | 941 | 2017 | Brazil: Sao Paulo | Ear |
| VIM-2 | NZ_NWBW01000093.1 | 1.7e-152 | 530.0 | *Pseudomonas aeruginosa* | ICBSVIM-2 | 947 | 2017 | Brazil: Sao Paulo | Environmental swab |
| VIM-2 | NZ_CP010893.1 | 6.4e-151 | 530.0 | *Pseudomonas sp.* | MRSN12121 | 36379 | NA | USA | Urine |
| VIM-2 | NZ_BCBD01000273.1 | 6.9e-152 | 529.6 | *Pseudomonas sp.* | NBRC 111127 | 3009 | NA | NA | NA |
| VIM-2 | NZ_BCBE01000104.1 | 3.1e-151 | 530.0 | *Pseudomonas sp.* | NBRC 111128 | 17527 | NA | NA | NA |
| VIM-2 | NZ_CVVA01000017.1 | 1.8e-152 | 530.0 | *Pseudomonas aeruginosa* | P10_London_6_VIM_2_09_09 | 1022 | NA | NA | NA |
| VIM-2 | NZ_CVVH01000182.1 | 1.6e-151 | 530.0 | *Pseudomonas aeruginosa* | P11_London_17_VIM_2_10_09 | 9036 | NA | NA | NA |
| VIM-2 | NZ_CVUP01000182.1 | 2.6e-151 | 530.0 | *Pseudomonas aeruginosa* | P12_London_17_VIM_2_11_09 | 15035 | NA | NA | NA |
| VIM-2 | NZ_CVUL01000160.1 | 2.6e-151 | 530.0 | *Pseudomonas aeruginosa* | P13_London_14_VIM_2_12_09 | 15035 | NA | NA | NA |
| VIM-2 | NZ_CVVT01000092.1 | 2.7e-152 | 530.0 | *Pseudomonas aeruginosa* | P14_London_17_VIM_2_01_10 | 1514 | NA | NA | NA |
| VIM-2 | NZ_CVVD01000036.1 | 1.8e-152 | 530.0 | *Pseudomonas aeruginosa* | P16_Lonon_17_VIM_2_02_10 | 1012 | NA | NA | NA |
| VIM-2 | NZ_CVUV01000039.1 | 1.1e-151 | 530.0 | *Pseudomonas aeruginosa* | P17_North_West_14_VIM_2_03_10 | 6417 | NA | NA | NA |
| VIM-2 | NZ_CVVY01000315.1 | 2.7e-152 | 530.0 | *Pseudomonas aeruginosa* | P18_London_17_VIM_2_04_10 | 1524 | NA | NA | NA |
| VIM-2 | NZ_CVVU01000098.1 | 2.9e-152 | 530.0 | *Pseudomonas aeruginosa* | P19_London_7_VIM_2_05_10 | 1625 | NA | NA | NA |
| VIM-2 | NZ_CVUO01000054.1 | 1.8e-152 | 530.0 | *Pseudomonas aeruginosa* | P20_London_17_VIM_2_05_10 | 1018 | NA | NA | NA |
| VIM-2 | NZ_CVVE01000152.1 | 2.6e-151 | 530.0 | *Pseudomonas aeruginosa* | P21_London_17_VIM_2_06_10 | 15035 | NA | NA | NA |
| VIM-2 | NZ_CVVI01000002.1 | 1.0e-151 | 530.0 | *Pseudomonas aeruginosa* | P22_London_17_VIM_2_06_10 | 5825 | NA | NA | NA |
| VIM-2 | NZ_CVUR01000052.1 | 1.8e-152 | 530.0 | *Pseudomonas aeruginosa* | P24_London_17_VIM_2_08_10 | 1032 | NA | NA | NA |
| VIM-2 | NZ_CVVL01000058.1 | 1.8e-152 | 530.0 | *Pseudomonas aeruginosa* | P25_London_12_VIM_2_08_10 | 1020 | NA | NA | NA |
| VIM-2 | NZ_CVVN01000218.1 | 2.6e-151 | 530.0 | *Pseudomonas aeruginosa* | P26_Wales_1_VIM_2_11_10 | 15035 | NA | NA | NA |
| VIM-2 | NZ_CVVX01000292.1 | 2.6e-151 | 530.0 | *Pseudomonas aeruginosa* | P27_Wales_1_VIM_2_02_11 | 15035 | NA | NA | NA |
| VIM-2 | NZ_CVVO01000009.1 | 1.5e-151 | 530.0 | *Pseudomonas aeruginosa* | P29_London_12_VIM_2_05_11 | 8275 | NA | NA | NA |
| VIM-2 | NZ_CVVV01000124.1 | 2.8e-152 | 530.0 | *Pseudomonas aeruginosa* | P3_North_West_16_VIM_2_07_06 | 1571 | NA | NA | NA |
| VIM-2 | NZ_CVVK01000198.1 | 2.6e-151 | 530.0 | *Pseudomonas aeruginosa* | P30_South_East_2_VIM_2_10_11 | 15035 | NA | NA | NA |
| VIM-2 | NZ_CVVS01000315.1 | 2.6e-151 | 530.0 | *Pseudomonas aeruginosa* | P31_Wales_1_VIM_2_11_11 | 15035 | NA | NA | NA |
| VIM-2 | NZ_CVVJ01000185.1 | 2.6e-151 | 530.0 | *Pseudomonas aeruginosa* | P32_London_17_VIM_2_10_11 | 15035 | NA | NA | NA |
| VIM-2 | NZ_CVVG01000173.1 | 2.6e-151 | 530.0 | *Pseudomonas aeruginosa* | P33_London_28_VIM_2_02_12 | 15035 | NA | NA | NA |
| VIM-2 | NZ_CVVF01000171.1 | 2.6e-151 | 530.0 | *Pseudomonas aeruginosa* | P34_London_28_VIM_2_02_12 | 15035 | NA | NA | NA |
| VIM-2 | NZ_CVUS01000017.1 | 2.5e-151 | 530.0 | *Pseudomonas aeruginosa* | P35_London_26_VIM_2_05_12 | 14212 | NA | NA | NA |
| VIM-2 | NZ_CVVP01000224.1 | 2.6e-151 | 530.0 | *Pseudomonas aeruginosa* | P36_West_Midlands_5_VIM_2_06_12 | 15035 | NA | NA | NA |
| VIM-2 | NZ_CVUJ01000038.1 | 1.8e-152 | 530.0 | *Pseudomonas aeruginosa* | P37_London_28_VIM_2_07_12 | 1043 | NA | NA | NA |
| VIM-2 | NZ_CVUT01000041.1 | 1.8e-152 | 530.0 | *Pseudomonas aeruginosa* | P38_London_12_VIM_2_08_12 | 1018 | NA | NA | NA |
| VIM-2 | NZ_CVWA01000091.1 | 2.7e-152 | 530.0 | *Pseudomonas aeruginosa* | P4_London_1_VIM_2_10_07 | 1524 | NA | NA | NA |
| VIM-2 | NZ_CVUM01000027.1 | 1.8e-152 | 530.0 | *Pseudomonas aeruginosa* | P40_Scotland_4_VIM_2_09_12 | 1026 | NA | NA | NA |
| VIM-2 | NZ_CVWH01000032.1 | 1.8e-152 | 530.0 | *Pseudomonas aeruginosa* | P42_1_London_26_VIM_2_10_12 | 1020 | NA | NA | NA |
| VIM-2 | NZ_CVWI01000150.1 | 2.6e-151 | 530.0 | *Pseudomonas aeruginosa* | P42_2_London_28_VIM_2_11_12 | 15035 | NA | NA | NA |
| VIM-2 | NZ_CVWJ01000041.1 | 1.8e-152 | 530.0 | *Pseudomonas aeruginosa* | P43_1_London_9_VIM_2_11_12 | 1022 | NA | NA | NA |
| VIM-2 | NZ_CVWK01000175.1 | 2.6e-151 | 530.0 | *Pseudomonas aeruginosa* | P43_2_London_9_VIM_2_11_12 | 15035 | NA | NA | NA |
| VIM-2 | NZ_CVWL01000379.1 | 2.6e-151 | 530.0 | *Pseudomonas aeruginosa* | P44_Wales_1_VIM_2_11_12 | 15035 | NA | NA | NA |
| VIM-2 | NZ_CVWO01000426.1 | 2.7e-152 | 530.0 | *Pseudomonas aeruginosa* | P45_London_17_VIM_2_12_12 | 1516 | NA | NA | NA |
| VIM-2 | NZ_CVWN01000191.1 | 6.9e-152 | 530.0 | *Pseudomonas aeruginosa* | P47_London_12_VIM_2_12_12 | 3897 | NA | NA | NA |
| VIM-2 | NZ_CVWT01000424.1 | 7.0e-152 | 530.0 | *Pseudomonas aeruginosa* | P48_London_17_VIM_2_01_13 | 3984 | NA | NA | NA |
| VIM-2 | NZ_CVWQ01000099.1 | 1.4e-150 | 530.0 | *Pseudomonas aeruginosa* | P49_London_7_VIM_2_01_13 | 79069 | NA | NA | NA |
| VIM-2 | NZ_CVWS01000440.1 | 2.1e-152 | 530.4 | *Pseudomonas aeruginosa* | P5_London_26_VIM_2_01_09 | 1572 | NA | NA | NA |
| VIM-2 | NZ_CVWP01000175.1 | 2.6e-151 | 530.0 | *Pseudomonas aeruginosa* | P50_London_9_VIM_2_01_13 | 15035 | NA | NA | NA |
| VIM-2 | NZ_CVWR01000046.1 | 2.6e-151 | 530.0 | *Pseudomonas aeruginosa* | P51_1_London_9_VIM_2_02_13 | 15035 | NA | NA | NA |
| VIM-2 | NZ_CVWU01000051.1 | 1.8e-152 | 530.0 | *Pseudomonas aeruginosa* | P51_2_London_11_VIM_2_02_13 | 1027 | NA | NA | NA |
| VIM-2 | NZ_CVWV01000016.1 | 2.6e-151 | 530.0 | *Pseudomonas aeruginosa* | P52_1_London_26_VIM_2_02_13 | 15035 | NA | NA | NA |
| VIM-2 | NZ_CVWW01000030.1 | 2.6e-151 | 530.0 | *Pseudomonas aeruginosa* | P52_2_London_26_VIM_2_02_13 | 15035 | NA | NA | NA |
| VIM-2 | NZ_CWER01000165.1 | 8.2e-152 | 530.0 | *Pseudomonas aeruginosa* | P53_London_9_VIM_2_02_13 | 4645 | NA | NA | NA |
| VIM-2 | NZ_CWFR01000312.1 | 1.0e-151 | 530.0 | *Pseudomonas aeruginosa* | P54_1_London_24_VIM_2_04_13 | 5826 | NA | NA | NA |
| VIM-2 | NZ_CVXL01000023.1 | 2.4e-151 | 530.0 | *Pseudomonas aeruginosa* | P54_2_London_24_VIM_2_04_13 | 13396 | NA | NA | NA |
| VIM-2 | NZ_CVWX01000168.1 | 2.6e-151 | 530.0 | *Pseudomonas aeruginosa* | P55_London_26_VIM_2_05_13 | 15035 | NA | NA | NA |
| VIM-2 | NZ_CVXC01000069.1 | 1.8e-152 | 530.0 | *Pseudomonas aeruginosa* | P56_London_12_VIM_2_07_13 | 1020 | NA | NA | NA |
| VIM-2 | NZ_CVWZ01000040.1 | 1.8e-152 | 530.0 | *Pseudomonas aeruginosa* | P57_South_East_3_VIM_2_09_13 | 1040 | NA | NA | NA |
| VIM-2 | NZ_CVXN01000005.1 | 2.6e-151 | 530.0 | *Pseudomonas aeruginosa* | P59_Wales_1_VIM_2_09_13 | 15035 | NA | NA | NA |
| VIM-2 | NZ_CVXD01000052.1 | 1.8e-152 | 530.0 | *Pseudomonas aeruginosa* | P60_London_6_VIM_2_11_13 | 1042 | NA | NA | NA |
| VIM-2 | NZ_CVXB01000200.1 | 2.6e-151 | 530.0 | *Pseudomonas aeruginosa* | P61_London_9_VIM_2_11_13 | 15035 | NA | NA | NA |
| VIM-2 | NZ_CWEO01000045.1 | 2.5e-151 | 530.0 | *Pseudomonas aeruginosa* | P62_London_9_VIM_2_01_14 | 14204 | NA | NA | NA |
| VIM-2 | NZ_CWFH01000038.1 | 2.3e-151 | 530.0 | *Pseudomonas aeruginosa* | P63_London_25_VIM_2_03_14 | 12782 | NA | NA | NA |
| VIM-2 | NZ_CVXP01000128.1 | 1.8e-152 | 530.0 | *Pseudomonas aeruginosa* | P7_London_17_VIM_2_06_09 | 1002 | NA | NA | NA |
| VIM-2 | NZ_CWET01000138.1 | 1.8e-152 | 530.0 | *Pseudomonas aeruginosa* | P8_1_South_East_10_VIM_2_07_09 | 999 | NA | NA | NA |
| VIM-2 | NZ_CVXH01000194.1 | 2.6e-151 | 530.0 | *Pseudomonas aeruginosa* | P8_2_London_17_VIM_2_07_13 | 15035 | NA | NA | NA |
| VIM-2 | NZ_AOIH01000060.1 | 3.7e-152 | 530.0 | *Pseudomonas aeruginosa* | PA21_ST175 | 2117 | 2010 | Spain | Blood |
| VIM-2 | NZ_CP017293.1 | 1.2e-148 | 530.0 | *Pseudomonas aeruginosa* | PA83 | 6816227 | 2013 | Germany | NA |
| VIM-2 | NZ_CP017073.1 | 8.1e-149 | 530.4 | *Pseudomonas putida* | PP112420 | 6031212 | 2011 | China: Guangzhou | Urine |
| VIM-2 | NZ_CP016955.1 | 1.3e-148 | 530.0 | *Pseudomonas aeruginosa* | RIVM-EMC2982 | 7380063 | NA | Netherlands: Bilthoven | NA |
| VIM-2 | NZ_CP011370.1 | 2.8e-150 | 530.0 | *Pseudomonas aeruginosa* | S04 90 | 159187 | 2013 | Netherlands: Rotterdam | Microbial material |
| VIM-2 | NZ_NFGK01000057.1 | 2.4e-151 | 530.0 | *Pseudomonas aeruginosa* | S422_C09_BS | 13810 | 2013/2014 | Italy: Udine | Blood |
| VIM-2 | NZ_NFGJ01000105.1 | 1.8e-152 | 530.0 | *Pseudomonas aeruginosa* | S426_C09_BS | 1036 | 2013/2014 | Italy: Udine | Blood |
| VIM-2 | NZ_NFGI01000040.1 | 2.4e-151 | 530.0 | *Pseudomonas aeruginosa* | S432_C09_RS | 13810 | 2013/2014 | Italy: Udine | Respiratory Sample |
| VIM-2 | NZ_NFGH01000056.1 | 2.4e-151 | 530.0 | *Pseudomonas aeruginosa* | S434_C09_BS | 13810 | 2013/2014 | Italy: Udine | Blood |
| VIM-2 | NZ_NFGG01000040.1 | 2.4e-151 | 530.0 | *Pseudomonas aeruginosa* | S435_C09_BS | 13710 | 2013/2014 | Italy: Udine | Blood |
| VIM-2 | NZ_NFGF01000042.1 | 2.4e-151 | 530.0 | *Pseudomonas aeruginosa* | S440_C09_BS | 13710 | 2013/2014 | Italy: Udine | Blood |
| VIM-2 | NZ_NFGE01000042.1 | 2.4e-151 | 530.0 | *Pseudomonas aeruginosa* | S442_C09_BS | 13710 | 2013/2014 | Italy: Udine | Blood |
| VIM-2 | NZ_NFGD01000045.1 | 2.4e-151 | 530.0 | *Pseudomonas aeruginosa* | S443_C09_RS | 13710 | 2013/2014 | Italy: Udine | Respiratory Sample |
| VIM-2 | NZ_NFGA01000029.1 | 1.7e-150 | 530.0 | *Pseudomonas aeruginosa* | S558_C10_BS | 97921 | 2013/2014 | Italy: Firenze | Blood |
| VIM-2 | NZ_NFGT01000092.1 | 2.0e-152 | 530.0 | *Pseudomonas aeruginosa* | S61_C01_BS | 1111 | 2013/2014 | Italy: Milano | Blood |
| VIM-2 | NZ_NFFR01000057.1 | 3.9e-152 | 530.0 | *Pseudomonas aeruginosa* | S669_C14_BS | 2201 | 2013/2014 | Italy: Roma | Blood |
| VIM-2 | NZ_NFFP01000094.1 | 4.6e-152 | 530.0 | *Pseudomonas aeruginosa* | S708_C14_RS | 2606 | 2013/2014 | Italy: Roma | Respiratory Sample |
| VIM-2 | NZ_NFFN01000017.1 | 2.6e-150 | 530.0 | *Pseudomonas aeruginosa* | S749_C15_RS | 146236 | 2013/2014 | Italy: San Giovanni Rotondo | Respiratory Sample |
| VIM-2 | NZ_NFFJ01000075.1 | 1.1e-151 | 530.4 | *Pseudomonas aeruginosa* | S794_C17_BS | 8276 | 2013/2014 | Italy: Napoli | Blood |
| VIM-2 | NZ_NFFH01000037.1 | 6.2e-151 | 530.4 | *Pseudomonas aeruginosa* | S819_C17_BS | 45581 | 2013/2014 | Italy: Napoli | Blood |
| VIM-2 | NZ_NFFF01000041.1 | 6.2e-151 | 530.4 | *Pseudomonas aeruginosa* | S827_C17_BS | 45581 | 2013/2014 | Italy: Napoli | Blood |
| VIM-2 | NZ_NFFD01000041.1 | 6.2e-151 | 530.4 | *Pseudomonas aeruginosa* | S830_C17_BS | 45581 | 2013/2014 | Italy: Napoli | Blood |
| VIM-2 | NZ_LYTW01000096.1 | 8.0e-152 | 530.0 | *Pseudomonas aeruginosa* | TRN6633 | 4566 | 2013 | Russia | NA |
| VIM-2 | NZ_LYTX01000091.1 | 8.0e-152 | 530.0 | *Pseudomonas aeruginosa* | TRN6635 | 4563 | 2013 | Russia | NA |
| VIM-2 | NZ_CP008739.1 | 9.2e-149 | 530.4 | *Pseudomonas aeruginosa* | VRFPA04 | 6818030 | 2013 | India:Chennai | Corneal button from patient with corneal keratitis |
| VIM-2 | NZ_MBPN01000207.1 | 6.5e-152 | 530.0 | *Pseudomonas sp.* | WCHP16 | 3690 | 2015 | China: Sichuan | Sewage |
| VIM-2 | NZ_LLVI01000133.1 | 2.7e-152 | 530.0 | *Pseudomonas aeruginosa* | WH-SGI-V-07322 | 1508 | 2003 | Colombia | Blood |
| VIM-2 | NZ_LLVL01000137.1 | 2.7e-152 | 530.0 | *Pseudomonas aeruginosa* | WH-SGI-V-07325 | 1508 | 1999 | Colombia | Urine |
| VIM-2 | NZ_LLQC01000091.1 | 3.8e-152 | 530.0 | *Pseudomonas aeruginosa* | WH-SGI-V-07496 | 2165 | 2005 | USA | Hospital |
| VIM-2 | NZ_LLQZ01000051.1 | 1.3e-150 | 530.0 | *Pseudomonas aeruginosa* | WH-SGI-V-07628 | 76200 | 2005 | USA | Hospital |
| VIM-36 | NZ_LLSF01000116.1 | 3.4e-152 | 530.4 | *Pseudomonas aeruginosa* | WH-SGI-V-07685 | 2498 | 2005 | USA | Hospital |
| VIM-4 | NZ_MPBV01000001.1 | 1.7e-149 | 531.6 | *Pseudomonas aeruginosa* | AR_0054 | 2863301 | NA | NA | NA |
| VIM-4 | NZ_JTWZ01000116.1 | 1.9e-152 | 531.6 | *Pseudomonas aeruginosa* | AZPAE14706 | 3202 | 2012 | Greece | Intra-abdominal tract infection |
| VIM-5 | NZ_JTZJ01000113.1 | 2.9e-152 | 529.3 | *Pseudomonas aeruginosa* | AZPAE13860 | 952 | 2010 | India | NA |
| VIM-5 | NZ_JTTX01000070.1 | 8.6e-152 | 529.3 | *Pseudomonas aeruginosa* | AZPAE14865 | 2861 | 2007 | India: Chennai | Respiratory tract infection |
| VIM-5 | NZ_JTSP01000126.1 | 5.3e-152 | 529.3 | *Pseudomonas aeruginosa* | AZPAE14900 | 1760 | 2008 | India: Chennai | Intra-abdominal tract infection |
| VIM-5 | NZ_PJCP01000041.1 | 4.1e-151 | 529.3 | *Pseudomonas guariconensis* | MR119 | 13479 | 2014 | Nigeria: Ibadan | Soil |
| VIM-5 | NZ_PJCL01000002.1 | 1.5e-149 | 529.3 | *Pseudomonas plecoglossicida* | MR134 | 491336 | 2014 | Nigeria: Ibadan | Soil |
| VIM-5 | NZ_PJCL01000056.1 | 9.5e-152 | 529.3 | *Pseudomonas plecoglossicida* | MR134 | 3169 | 2014 | Nigeria: Ibadan | Soil |
| VIM-5 | NZ_PJCM01000001.1 | 1.5e-149 | 529.3 | *Pseudomonas plecoglossicida* | MR135 | 491336 | 2014 | Nigeria: Ibadan | Soil |
| VIM-5 | NZ_PJCQ01000042.1 | 3.2e-151 | 529.3 | *Pseudomonas guariconensis* | MR144 | 10655 | 2014 | Nigeria: Ibadan | Soil |
| VIM-5 | NZ_PJCR01000038.1 | 4.1e-151 | 529.3 | *Pseudomonas guariconensis* | MR149 | 13479 | 2014 | Nigeria: Ibadan | Soil |
| VIM-5 | NZ_PJCO01000068.1 | 2.4e-151 | 529.3 | *Pseudomonas plecoglossicida* | MR170 | 7887 | 2014 | Nigeria: Ibadan | Soil |
| VIM-5 | NZ_PJCJ01000004.1 | 1.5e-149 | 529.3 | *Pseudomonas plecoglossicida* | MR69 | 491416 | 2014 | Nigeria: Ibadan | Soil |
| VIM-5 | NZ_PJCK01000003.1 | 1.5e-149 | 529.3 | *Pseudomonas plecoglossicida* | MR70 | 491062 | 2014 | Nigeria: Ibadan | Soil |
| VIM-5 | NZ_PJCN01000069.1 | 2.4e-151 | 529.3 | *Pseudomonas plecoglossicida* | MR83 | 7887 | 2014 | Nigeria: Ibadan | Soil |
| VIM-6 | NZ_LOHJ01000053.1 | 1.1e-150 | 528.9 | *Pseudomonas aeruginosa* | 105857 | 28969 | 2008 | USA: Fort Sam Houston | Right knee |
| VIM-6 | NZ_JYGB02000018.1 | 1.2e-150 | 528.9 | *Pseudomonas aeruginosa* | MRSN 17623 | 30188 | NA | USA | NA |

NA, not available.
