# Supplementary material for: Carbapenemases on the move: it’s good to be on ICEs
Source: Mob DNA. 2018 Dec 19;9:37. doi: 10.1186/s13100-018-0141-4 (PMC6299553; doi:10.1186/s13100-018-0141-4)
Supplement: Supplementary file 2 — Figure S1. Genetic environment of a novel genomic island (GI) harboring blaVIM-2 in P. aeruginosa strain AZPAE13853. Gene cassettes are shown by pale blue boxes, the conserved sequence (5’-CS) of the integron as orange boxes. Gaps > 50 bp are indicated by dashed red lines and the length in bp given. Transposons IRs are shown as flags, with the flat side at the outer boundary of the transposon. (DOCX 34 kb) [file 13100_2018_141_MOESM2_ESM.docx]

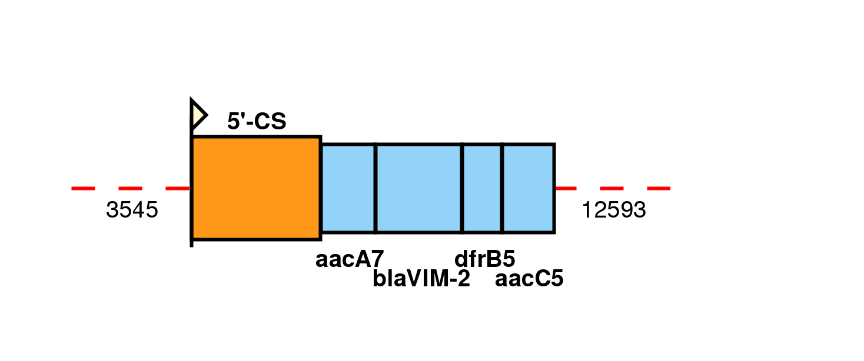


**Figure S1**. Genetic environment of a novel genomic island (GI) harboring *bla*_VIM-2_ in *P. aeruginosa* strain AZPAE13853. Gene cassettes are shown by pale blue boxes, the conserved sequence (5’-CS) of the integron as orange boxes. Gaps >50 bp are indicated by dashed red lines and the length in bp given. Transposons IRs are shown as flags, with the flat side at the outer boundary of the transposon.
